# The Effects of Media Reports on Disease Spread and Important Public Health Measurements

**DOI:** 10.1371/journal.pone.0141423

**Published:** 2015-11-03

**Authors:** Shannon Collinson, Kamran Khan, Jane M. Heffernan

**Affiliations:** 1 Modelling Infection and Immunity Lab, Centre for Disease Modelling, York University, Toronto, Canada; 2 Mathematics & Statistics, York University, Toronto, Canada; 3 Li Ka Shing Knowledge Institute, St. Michael’s Hospital, Toronto, Canada; 4 Department of Medicine, Division of Infectious Diseases, University of Toronto, Toronto, Canada; University of Waterloo, CANADA

## Abstract

Controlling the spread of influenza to reduce the effects of infection on a population is an important mandate of public health. Mass media reports on an epidemic or pandemic can provide important information to the public, and in turn, can induce positive healthy behaviour practices (i.e., handwashing, social distancing) in the individuals, that will reduce the probability of contracting the disease. Mass media fatigue, however, can dampen these effects. Mathematical models can be used to study the effects of mass media reports on epidemic/pandemic outcomes. In this study we employ a stochastic agent based model to provide a quantification of mass media reports on the variability in important public health measurements. We also include mass media report data compiled by the Global Public Health Intelligence Network, to study the effects of mass media reports in the 2009 H1N1 pandemic. We find that the report rate and the rate at which individuals relax their healthy behaviours (media fatigue) greatly affect the variability in important public health measurements. When the mass media reporting data is included in the model, two peaks of infection result.

## Introduction

Controlling the spread of influenza to reduce the effects of infection on a population is an important mandate of public health. Influenza control can be achieved through various strategies including vaccination, the use of drug therapy, hand washing and social distancing—removing oneself as much as possible from the population [1, 2]. Vaccines and drug therapies, however, may be ineffective (e.g. resistance to antivirals) or unavailable (i.e. pending development of a new vaccine). Thus, hand washing and social distancing practices (i.e., ranging from moderate to extreme total isolation), which can be used at all times during an infectious disease outbreak are integral in decreasing the probability of contracting and transmitting infection [1].

Mass media campaigns can be used to provide information on current and effective vaccination, drug therapy and social distancing measures [1]. Public health education campaigns, that include informative literature (i.e., pamphlets), posters, newspaper articles and advertisements, radio and television messages, and social media outlets (i.e., twitter, facebook) are used daily to inform the public on current health issues. Mass media outlets can aid in dissemination of this information. Studies of mass media campaigns and healthy behaviour have reported that mass media campaigns can elicit positive behaviour change and even prevent negative behaviour change in individuals [3–9]. It is therefore concluded that mass media campaigns should be used to inform the public so that behaviour change can result [3–10]. It has been shown that information conveyed by the media is becoming the critical factor as to whether or not a vaccination campaign will succeed [11]. It is believed that the media coverage of the recent SARS and 2009 H1N1 epidemics had an effect on the total spread of these pathogens [4, 7, 12, 13]. More recently, MERS-CoV, Ebola, and H7N9 have been the subject of many media reports.

Pervasive media coverage of social problems may lead to desensitization to media reports [14–16]: a diminished emotional responsiveness to a negative or an aversive stimulus after repeated exposure. This same phenomenon can occur with health events –individuals may be more likely to take precautions against becoming ill with the first reports of an epidemic or health threat, but individual sensitivity to disease reports may diminsh with time, and social distancing practices may be relaxed [15, 17–19], affecting disease transmission. To better control infectious diseases a better understanding of the effects of mass media on the uptake and waning of social distancing practices is needed.

Mathematical models can be employed to study the effects of media in an epidemic [19–32]. For example, the effects of media reports (i.e., on disease incidence, hospitalizations, deaths) can be incorporated as a function *f*(*S*, *E*, *I*, *R*) that directly affects the transmission rate in a susceptible-exposed-infected-recovered (SEIR) epidemiological model [19–27, 32, 33]:

$$\begin{array}{c} \begin{array}{ccc} \dot{S} & = & \lambda -f(S,E,I,R)\beta SI-dS\\ \dot{E} & = & f(S,E,I,R)\beta SI-\sigma E-dE\\ \dot{I} & = & \sigma E-\gamma I-dI\\ \dot{R} & = & \gamma I-dR\;. \end{array} \end{array}$$ (1)

This model has provided the basis of the vast majority of past mathematical modelling studies of media during an epidemic. In a previous study on the effects of mass media reports on important public health measures (i.e., peak number of infections, time of peak infection, end of epidemic and total number of infections), we demonstrated that the variability in these measures can vary drastically depending on the media function *f*(*S*, *E*, *I*, *R*) chosen [33]. In this study, we then demonstrated that the recommended public health measures to mitigate epidemic outcomes were not consistent over different choices of *f*(*S*, *E*, *I*, *R*), and we suggested that models that include a mass media compartment, that better represents population interactions with mass media, should be considered [33]. For example,

$$\begin{array}{c} \begin{array}{ccc} \dot{S} & = & \lambda -\beta SI-dS-g(M)S\\ {\dot{S}}_{M} & = & g\left(M\right)S-dS_{M}\\ \dot{E} & = & \beta SI-\sigma E-dE\\ \dot{I} & = & \sigma E-\gamma I-dI\\ \dot{R} & = & \gamma I-dR\\ \dot{M} & = & f(S,E,I,R)-\mu M\;, \end{array} \end{array}$$ (2)

explicity includes a mass media compartment, allowing a sensitivity analysis over biological assumptions that determine the function *f*(*S*, *E*, *I*, *R*). Here, *g*(*M*) is a function representing the number of mass media reports *M*, *g*(*M*)*S* models the interaction that individuals have with media that can change their behaviour, *S*_*M*_ is a class of susceptibles that are media aware and are less susceptible to infection (or entirely protected through healthy and social distancing behaviours), and *μ* represents the effects of media fatigue. A further benefit of Eq (2) is that it enables the inclusion of mass media report data to replace *f*(*S*, *E*, *I*, *R*). Studies of the effects of mass media on epidemic outcomes employing systems of equations similar to Eq (2) exist in the literature [28–31], however, none of these studies have incorporated mass media data into their analysis. Also, quantification of the variability surrounding important public health measures has been ignored.

In this study we extend Eq (2) to include more levels of healthy behaviour, and vaccination. We then study the model in two different ways. First, we employ a stochastic agent-based simulation to study variability in key public health measurements. Second, we incorporate 2009 H1N1 mass media report data from the Global Public Health Intelligence Network (GPHIN) [34, 35] into the model and study the effects of this data and media fatigue on the pandemic dynamics in a closed population. From herewithin, we describe all healthy behaviours including isolation, limiting contacts and hand washing as ‘social distancing’. Social distancing levels included in the model are chosen to reflect different degrees of protection that healthy behaviour change can provide. Specifically, we include the extremes—no protection and total protection/isolation, and a moderate protection level. The uptake of social distancing behaviours, and vaccination are both assumed to be affected by mass media reports [3, 5, 6, 11].

In the following sections we motivate the model and determine the expected variability in key public health measurements (i.e., peak number of infections, time of peak infection, end of epidemic and total number of infections). A sensitivity analysis of the model is also completed so that parameters that most affect the key public health measurements can be determined. Interestingly, the model parameters representing mass media reports and media fatigue are identified. When mass media data [34] is incorporated into the deterministic model, a two-peak pandemic curve results. This curve has similar qualitative characteristics to that observed over the recent 2009 H1N1 pandemic.

## Methods

### Model

The model considers fully susceptible (*S*), social distancing susceptible (*S*_1_), isolated susceptible (*S*_2_), vaccinated (*V*), exposed (*E*), infectious (*I*) and recovered (*R*) populations. The media (*M*) is also incorporated as a separate compartment and affects the movement of individuals between the susceptible and vaccinated classes. The model is as follows:

$$\begin{array}{c} \begin{array}{ccc} \dot{S} & = & \lambda -\beta SI-\alpha SM-\nu SM+q_{1}S_{1}-dS\\ \dot{S_{1}} & = & -\beta_{1}S_{1}I+\alpha SM-\alpha_{1}S_{1}M-\nu_{1}S_{1}M+q_{2}S_{2}-q_{1}S_{1}-dS_{1}\\ \dot{S_{2}} & = & \alpha_{1}S_{1}M-q_{2}S_{2}-\nu_{2}S_{2}M-dS_{2}\\ \dot{V} & = & \nu SM+\nu_{1}S_{1}M+\nu_{2}S_{2}M-dV\\ \dot{E} & = & \beta SI+\beta_{1}S_{1}I-\sigma E-dE\\ \dot{I} & = & \sigma E-\gamma I-dI\\ \dot{R} & = & \gamma I-dR\\ \dot{M} & = & \rho \sigma E-\rho_{1}M\;. \end{array} \end{array}$$ (3)

**Fig 1:**
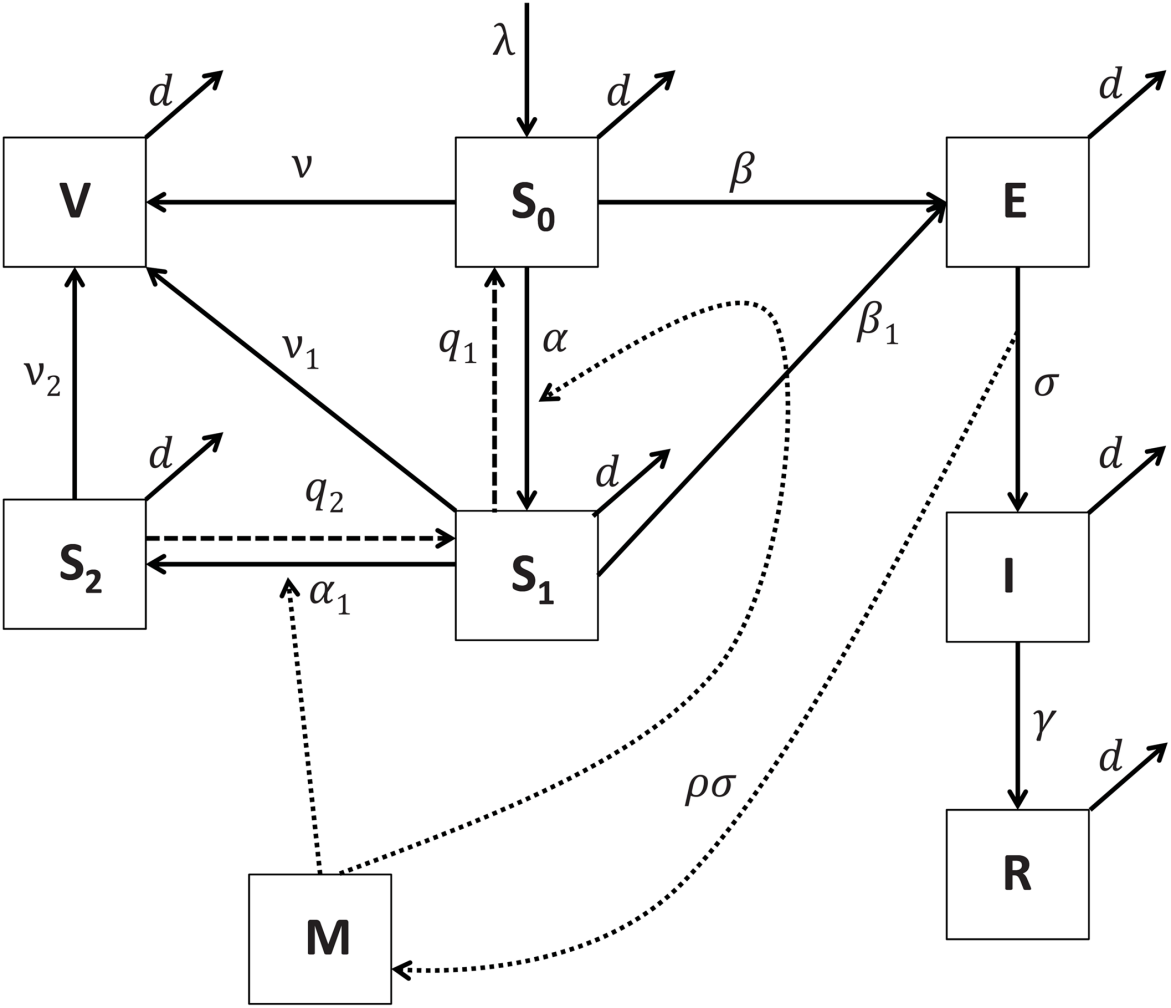
Model flow diagram. Flow diagram and schematic of the Agent Based Monte Carlo simulation.

A flow diagram of Eq (3) is shown in Fig 1. Briefly, fully susceptible individuals *S* can be infected (*βI*) and can take up precautionary measures motivated by interactions (listening, reading, watching) with media reports (*M*), such as vaccination (*ν*) or social distancing (*α*). Susceptible individuals in the social distancing class *S*_1_ can also be infected, but at a lower rate (*β*_1_), and can also take on precautionary measures such as vaccination (*ν*) and isolation (*α*_1_), motivated by exposure to media reports. It is assumed that isolated individuals (*S*_2_) are fully isolated and cannot be infected, but can be vaccinated (*ν*) as a result of exposure to media reports (*M*). It is assumed that vaccination provides more protection than any level of social distancing, thus, all vaccinated individuals move to the vaccinated class (*V*). Exposed individuals are produced via infection of susceptible individuals *S* and *S*_1_ and can become infectious (*σ*). Infectious individuals can recover from infection (*γ*). It is assumed that individuals are born (*λ*) fully susceptible and that social behaviour can wane (*q*_*i*_, *i* = 1, 2) which moves individuals in higher levels of social distancing to lower ones. It is also assumed that individuals can only die from natural causes (*d*) (a disease induced death rate is ignored).

An increase in media reports can be modelled using various different assumptions. Here, it is assumed that media reports are proportional to a fraction of incoming infectious cases *ρσE* that are symptomatic and/or reported to a doctor [36]. It is also assumed that the effects of mass media on social distancing behaviours can wane (*ρ*_1_) [14, 15, 37, 38]. The relaxation of social distancing behaviours *q*_1_ and *q*_2_ also reflect this behaviour.

Note that Eq (3) ignores covariances and higher order moments i.e. it assumes that all model classes are independent of all others. We include a derivation of Eq (3) in the Appendix that includes higher order moments. For the current study and parameter values, Eq (3) and the model provided in the Appendix do not differ significantly (not shown). Thus, we chose only to present results of Eq (3) here within.

Note that more levels of social distancing can be included in the susceptible populations. Social distancing can also be included in the exposed and infectious classes. For the purposes of the current study we chose to include only three levels of social distancing in the susceptible population—none, moderate, and total isolation. This selection has provided a more analytically tractable model to study mass media effects on social distancing and vaccination behaviours. Furthermore, an optimal number of social distancing levels that would best represent all social distancing behaviours in susceptible, exposed and infected populations is currently unknown. An extension of this model to study a ‘continuous social distance’ in all model populations, instead of discrete stages like that used here, is a course for future work.

### Stochastic model

Eq (3) is deterministic and cannot be used to study variability in infection. We have developed an Agent-based Monte Carlo (ABMC) simulation to study variability in key epidemic measurements when vaccination, social distancing, media and waning behaviour are included. The ABMC simulation models the population at the level of individual agents where each agent contains characteristics associated with infection status, birth, death and social distancing measures. The ABMC simulation moves forward in time using event times: the next time that an individual changes state within the system. To compare to a system of ordinary differential equations (ODEs) exponential distributions for all lifetimes are assumed. Table 1 lists the means of the lifetime distributions, corresponding to the parameters of Eq (3).

**Table 1:** Model classes and parameters. See text for more details.

Parameter	Definition	Value	ABMC	Ref
S(t)	Susceptible individuals		Time to move to *S*_1_	
			Time of vaccination	
*S*_1_(*t*)	Susceptible, social distancing practices		Time to move to *S*	
			Time to move to *S*_2_	
			Time of vaccination	
*S*_2_(*t*)	Susceptible, isolated		Time to move to *S*_1_	
			Time of vaccination	
E(t)	Exposed individuals		Time of progression to infectiousness	
I(t)	Infectious individuals		Recovery time	
			Time to infect a susceptible	
R(t)	Recovered individuals			
V(t)	Vaccinated individuals			
M(t)	Media reports			
*R*_0_	Basic reproductive ratio	1.3, 1.7		[39]
*β*_*i*_	Contact transmission rate for *S*_*i*_ class	3.712 × 10^−5^ (person-day)^−1^	Exponential distribution, mean $\frac{1}{\beta }$	calculated
*σ*	Transition rate from exposed to infectious	$\frac{1}{6-1/\gamma }$ day^−1^	Exponential distribution, mean $\frac{1}{\sigma }$	[39]
*γ*	Recovery rate	$\frac{1}{4}$ day^−1^	Exponential distribution, mean $\frac{1}{\gamma }$	[39]
*α*_*i*_	Social distancing uptake uptake rate for *S*_*i*_ class	0.04, 0.004 day^−1^	Exponential distribution, mean $\frac{1}{\alpha_{i}}$	assumed
*ν*_*i*_	Vaccination rate from *i* ^*th*^ socially distanced	10^−5^ − 0.002 day^−1^	Exponential distribution, mean $\frac{1}{\nu_{i}}$	[40]
*q*_*i*_	Relaxation rate of social distancing practices in *S*_*i*_	0.001 − 0.06 day^−1^	Exponential distribution, mean $\frac{1}{q}$	assumed
*ρ*	Fraction of infectious cases reported	0.01	Exponential distribution, mean $\frac{1}{\rho }$	
*ρ*_1_	Media waning rate	0.015 day^−1^	Exponential distribution, mean $\frac{1}{\rho_{1}}$	calculated

Fig 1 shows the different states that an individual can progress through during an epidemic. The attributes associated with each individual in each model class are listed in Table 1. Briefly, agents in each of the susceptible, exposed, infectious and recovered compartments are assigned event times corresponding to each event that allows an individual from that compartment to change state. To move froward in time the simulation chooses the minimum event time in the population and performs the corresponding event. It then searches for the next minimum event time and so on. The model events and mean event times are listed in Table 1.

### Parameter Values

Parameter values for Eq (3) can be found in Table 1. These values agree with the influenza literature and are further informed by the literature surrounding social behaviour responses to media reports.

It is assumed that individuals experience on average 6 days of infection (1/*σ* + 1/*γ*) and can transmit the infection for approximately 4 days at the end of the infection time period (1/*γ* = 4 days). The transmission rate of infection *β* is calculated using the basic reproductive ratio *R*_0_ where *R*_0_ takes the value 1.5 which lies in the reported range 1.3 to 1.7 [39]. When vaccination is included in the model, it is employed only during the second wave of infection as vaccination was not available during the first wave of the 2009 H1N1 pandemic. It is assumed that on average 25% to 40% of the population is vaccinated [40]. The social distancing uptake and relaxation rate values (*α*_*i*_ and *q*_*i*_) are assumed so that the pandemic curve has a similar magnitude and length to that observed for 2009 H1N1 in Canada [1, 2, 39–41].

**Fig 2:**
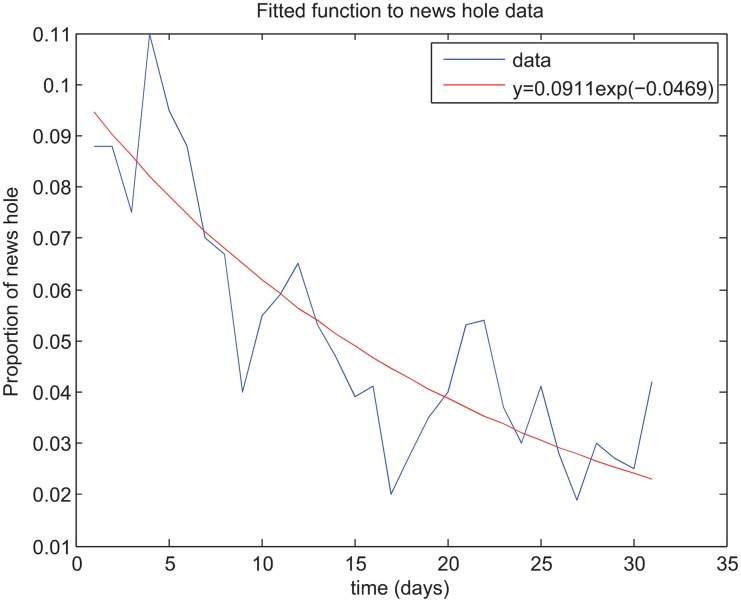
Media fatigue. Data describing media fatigue is shown (blue) [16]. An exponential curve is fit to the data to determine a media fatigue waning rate (red). The resulting equation is *f*(*t*) = 0.09911 exp − 0.0469*t*. Right panel: Media reports. Second wave of an epidemic produced from the fitted value for *ρ*_1_ from [16] and parameter values in [41]. The x-axis is time in days.

Fig 2 shows data extracted from a study on the ‘newsworthiness’ of influenza H1N1 as extracted from newspaper homepages across 12 websites from April 29 to May 28, 2009 [16]. The solid line on Fig 2 shows an exponential curve fit to the data points. It is assumed that the media waning rate *ρ*_1_ is equal to the decay rate of this exponential curve fit.

**Fig 3:**
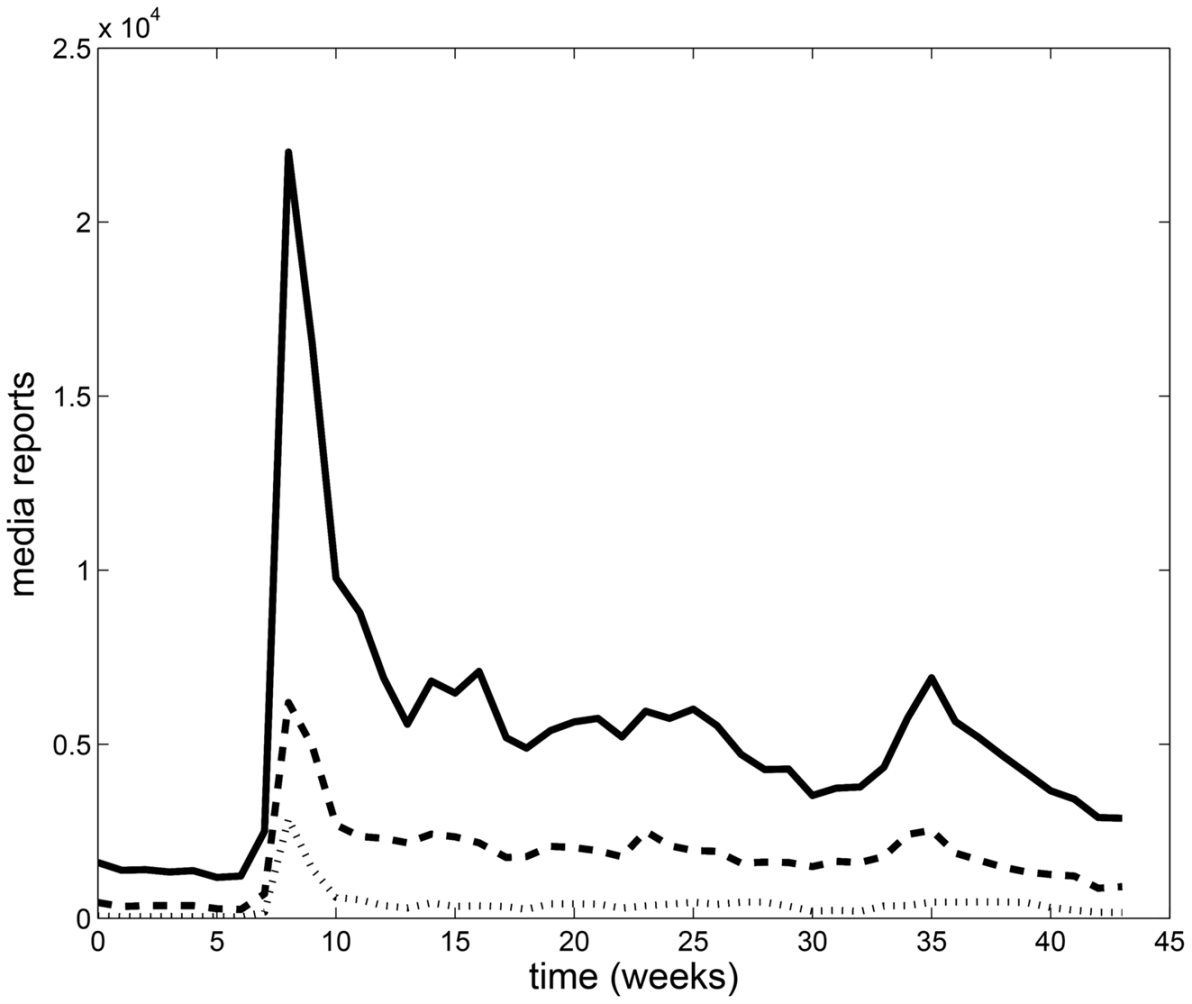
GPHIN media data. Media data collected by GPHIN from all media worldwide. The time scale is weeks with 0 corresponding to March 1, 2009. The solid line is all media data collected, the dotted line is French and English media data and the dashed line is English language media data.

The Government of Canada collects data on emerging public health threats through the Global Public Health Intelligence Network (GPHIN) [34]. Fig 3 shows the weekly number of news reports as collected by GPHIN from March 1 to December 27, 2009, broken down by language. This data is used to inform the media compartment *M* of Eq (3) in a study of the effects of mass media reports during the 2009 H1N1 pandemic.

## Results

### Disease free equilibrium (DFE)

The disease free equilibrium (DFE), or uninfected equilibrium, is the equilibrium point at which no infection exists in the system. The DFE for Eq (3) is

$$\begin{array}{c} E_{0}=\left(S,S_{1},S_{2},V,E,I,R,M\right)=\left(\frac{\lambda }{d},0,0,0,0,0,0,0\right)\;. \end{array}$$ (4)

and the total population *N* = *λ*/*d*.

### Basic Reproductive Ratio

The basic reproductive ratio, *R*_0_, is defined as the number of new infectious individuals produced by one infectious individual when introduced into a fully susceptible population. Generally, if *R*_0_ < 1, a disease will die out, but if *R*_0_ > 1, the pathogen will spread in the population. There are various methods that can be used to calculate *R*_0_ [42]. Using the survivor function and the next generation methods [42] to calculate *R*_0_ for Eq (3), we obtain

$$\begin{array}{c} R_{0}=\frac{\beta S_{0}}{\left(\gamma +d\right)}\frac{\sigma }{\left(\sigma +d\right)} \end{array}$$ (5)

where *S*_0_ = *λ*/*d* from *E*_0_. This is an intuitive result as it applies directly to the underlying processes of transmission and progression through the different stages of infection. Here, the first term corresponds to the production of an exposed individual through transmission of infection from an infected individual to a susceptible, and the second term gives the probability that a new exposed individual progresses to the infectious compartment. We note here, that the disease free equilibrium *E*_0_ is locally asymptotically stable if *R*_0_ < 1 and unstable if *R*_0_ > 1 (see Appendix for proof).

### Infected equilibrium

Eq (3) has at least one infected equilibrium if the following equations are satisfied.

$$\begin{array}{c} \begin{array}{ccc} V & = & \left(\nu S+\nu_{1}S_{1}+\nu_{2}S_{2}\right)\frac{\rho (\gamma +d)}{\rho_{1}d}I\\ E & = & \frac{\gamma +d}{\sigma }I\\ R & = & \frac{\gamma }{d}I\\ M & = & \frac{\rho (\gamma +d)}{\rho_{1}}I \end{array} \end{array}$$ (6)

where

$$\begin{array}{c} \begin{array}{ccc} S & = & \frac{\lambda }{dR_{0}}-\frac{\beta_{1}S_{1}}{\beta }\\ S_{2} & = & \frac{\alpha_{1}\rho \left(\gamma +d\right)}{q_{2}\rho_{1}+\nu_{2}\rho \gamma I+\nu_{2}\rho dI+d\rho_{1}}S_{1}I \end{array} \end{array}$$ (7)

and *I* and *S*_1_ satisfy

$$\begin{array}{c} \begin{array}{ccc} \lambda +q_{1}S_{1}-dS & = & (\beta (\frac{\lambda }{dR_{0}}-\frac{\beta_{1}S_{1}}{\beta })+\frac{\left(\alpha +\nu )\rho (\gamma +d\right)}{\rho 1}(\frac{\lambda }{dR_{0}}-\frac{\beta_{1}S_{1}}{\beta }))I\\ \frac{\alpha \rho \left(\gamma +d\right)S_{0}I}{R_{0}\rho_{1}} & = & (\beta_{1}I+\frac{\alpha \beta_{1}\rho I\left(\gamma +d\right)}{\beta \rho_{1}}+q_{1}+\left(\alpha_{1}+\nu_{1}\right)\frac{\rho I(\gamma +d)}{\rho_{1}}-\frac{q_{2}\alpha_{1}\rho I\left(\gamma +d\right)}{q_{2}\rho_{1}+\nu_{2}\rho I\gamma +\nu_{2}\rho Id+d\rho_{1}}+d)S_{1} \end{array} \end{array}$$ (8)

### Translation to short term epidemics

For short term epidemics such as influenza, births (*λ*) and natural death (*d*) can be ignored since the total number of births and deaths over the course of the epidemic is very small. In the following sections it is assumed that *λ* = 0 and *d* = 0. The DFE then becomes

$$\begin{array}{c} E_{0}=\left(N,0,0,0,0,0,0,0\right) \end{array}$$

and

$$\begin{array}{c} R_{0}=\frac{\beta N}{\gamma } \end{array}$$

where *N* is the total population size. There is no infected equilibrium for this reduced system.

### Numerical simulations

We considered various scenarios with and without vaccination, social distancing and waning behaviour. Table 2 lists the values of the key epidemic measurements—peak time, peak magnitude, epidemic end, total infected, total vaccinated—resulting from Eq (3) for various combinations of vaccine uptake, media social distancing levels and media waning: (a) social distancing and vaccination are not considered; (b) extending to include one level of social distancing (i.e., *α* > 0), with no waning in behavioural practices (i.e., *q*_1_ = 0 and *ρ*_1_ = 0) and vaccination (*ν* = 0); (c) extending to include vaccination (*ν* > 0); (d) adding an isolated class *S*_2_ (*α*_1_ > 0); (e) adding media media waning (*ρ*_1_ > 0) and movement between social distancing classes through the relaxation of social distancing practices (*q*, *q*_1_, *q*_2_ > 0). In general, these studies demonstrated that, as expected, vaccination uptake and social distancing practices will reduce the impact of an epidemic, and that waning behaviour will lessen these benefits.

**Table 2:** Simulation results. Each row has results for the ODE models and the ABMC for 100 simulations (mean and standard error). (a) Results for a standard SEIR model; (b) SS_1_EIRM, including *S*_1_ and media reports; (c) SS_1_VEIRM, including vaccination of *S* and *S*_1_; (d) SS_1_S_2_VEIRM, extending to include *S*_2_ and vaccination; (e) SS_1_S_2_VEIRMw, extending to include media waning.

Model	Peak Time (days)	Peak Magnitude (I)	Epidemic end (day)	Total infected (I)	Total vaccinated (V)
(a)	40.50	730.63	149.08	6066.50	N/A
	40.10 ± 16.77	719.57 ± 234.43	135.98 ± 83.03	6061.20 ± 81.77	N/A
(b)	40.82	704.03	151.81	5967.00	N/A
	41.60 ± 9.81	722.60 ± 17.34	187.50 ± 64.90	5838.23 ± 471.67	N/A
(c)	35.50	566.38	112.67	4183.60	3983.40
	36.80 ± 4.54	663.34 ± 204.32	113.20 ± 8.77	4313.20 ± 321.60	3874.50 ± 220.50
(d)	19.71	275.03	66.73	1402.48	3524.66
	22.24 ± 8.62	293.30 ± 57.92	61.82 ± 8.13	1536.70 ± 441.43	3811.60 ± 435.84
(e)	20.93	182.85	67.72	1403.30	3008.30
	21.11 ± 8.48	311.25 ± 55.78	64.99 ± 7.58	1586.30 ± 409.87	3418.90 ± 615.45

### Variability in infection

Variability in the key epidemic measurements, as calculated from 100 runs of the ABMC simulation are also shown in Table 2. Overall, the results suggest that public health programs should be implemented to consider time intervals of 10–50 days for peak infections, and 20–160 days for the end of the epidemic, depending on the control and social distancing being practiced. We also see that the variability in the peak magnitude, total number of infections, and total number vaccinated is affected by these practices—decreases and increases in variability (calculated using the coefficient of variation = (standard deviation)/mean) can be observed. It is interesting to note that the addition of public health control measures can increase the variability in the total number of infections and vaccinations. This may be due to the fact that individuals can move between social distancing classes many times during the epidemic, providing greater uncertainty in these outcomes of the epidemic. This will affect public health preparedness strategies. It is important to understand what drives this variability, so that public health programs can focus on certain attributes of an epidemic, so as to minimize the variation.

### Sensitivity Analysis

To determine which parameters most affect model the model outcomes, a sensitivity analysis employing Latin Hypercube Sampling (LHS) and Partial Rank Correlation Coefficients (PRCC) is performed. These methods are said to be the most efficient methods for performing sensitivity analysis with variation in multiple parameters simultaneously [43].

**Fig 4:**
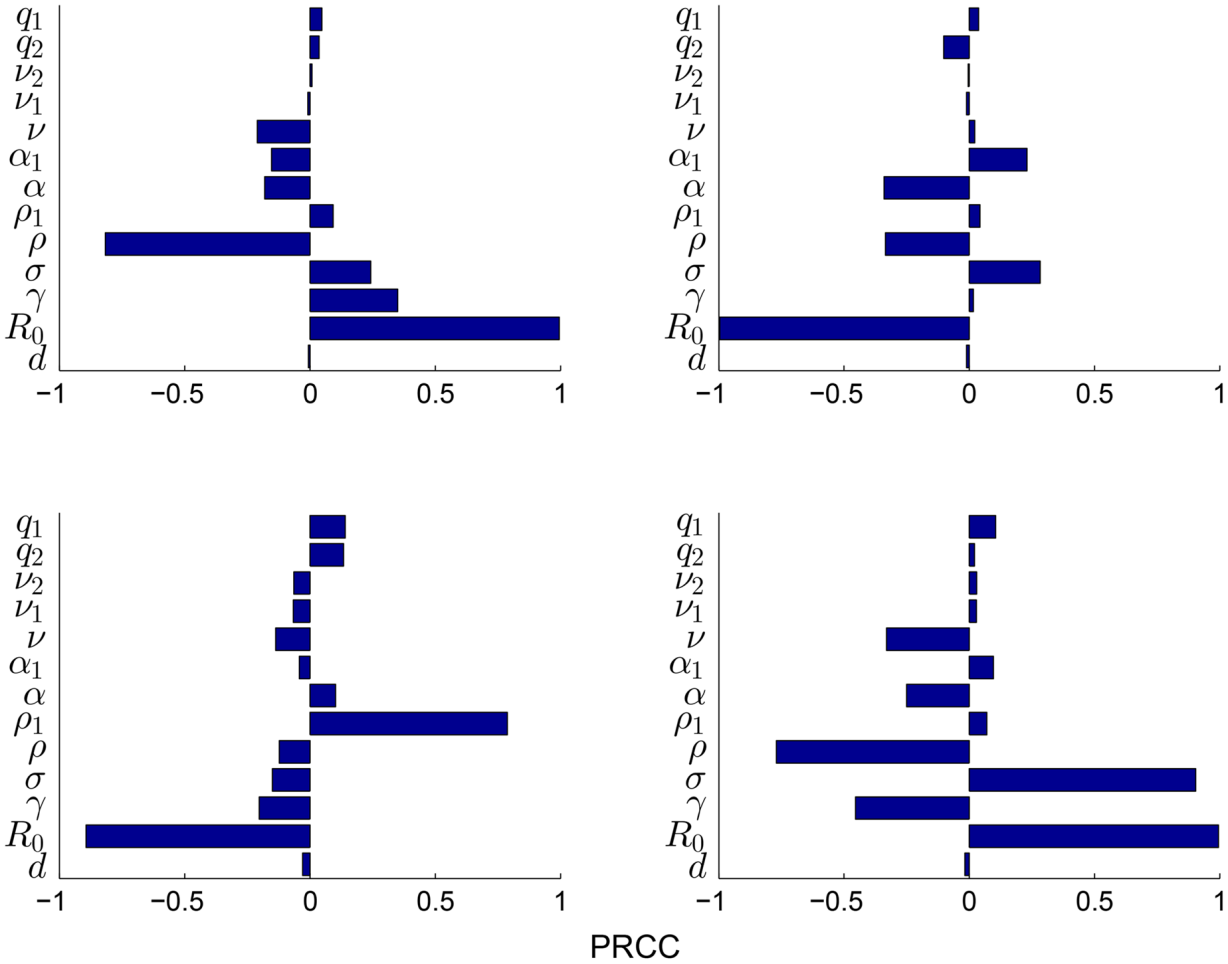
Sensitivity analysis. Partial rank correlation coefficients are shown for (a) Peak magnitude; (b) Peak time (c) End time; (d) Total number of infectious individuals. PRCC coefficients have negative or positive correlations to the public health outcomes of interest. The total population N is assumed to be constant.

The outcomes that are of interest for Eq (3) include the peak time of the epidemic (or time to the peak), the end time of the epidemic (or length of the epidemic), the peak magnitude of infectious and exposed individuals, and the total number of infectious individuals. Fig 4 shows the PRCC values for Eq (3) assuming a constant population size *N*. This figure shows that *R*_0_ is the most important parameter for each outcome. However, the total number of infections over an epidemic is also sensitive to changes in the rates at which individuals become infectious (*σ*) and recover (*γ*). The figure also shows that the peak magnitude and the total number of infections are both sensitive to the media parameter *ρ*, and the epidemic end time is sensitive to the media fatigue rate *ρ*_1_. Overall, the sensitivity analysis results show that to minimize the effect of the epidemic, public health programs should strive to (1) reduce transmission (effectively reducing *R*_0_), (2) effectively use drug therapies that can shorten the infectious period (effectively decreasing *σ* and increasing *γ*), (3) increase the number of reported cases *ρ*, and (4) provide educational programs throughout an epidemic that will help minimize the waning of positive social distancing practices *ρ*_1_. These results demonstrate that role of mass media can significantly affect the severity and length of an epidemic/pandemic. Thus, attention should be given to studying how best to use mass media during an epidemic, so that epidemic measurements can be minimized.

### Mass Media Data and Media Waning

**Fig 5:**
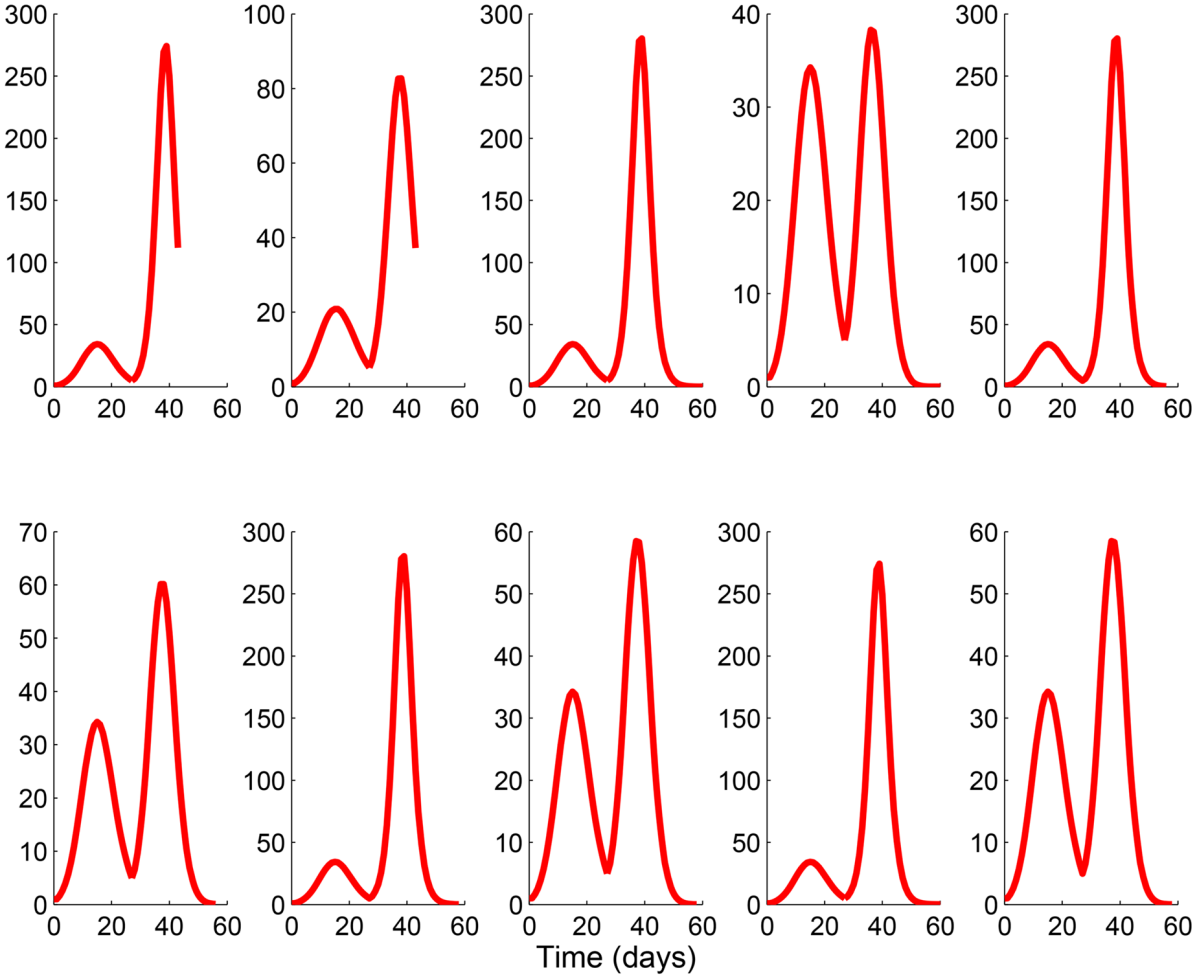
Infection curves with GPHIN data. In the left panel of this figure the pandemics do not have a vaccine available, in the right panel there is a vaccine available for the second wave of the pandemic. First row: influenza pandemic curve for the duration for which GPHIN has media data collected. Second row: media is kept constant at the level of the last weeks data collection after the end of data collection. Third row: media decays linearly after final data point and is then held at 0. Fourth row: no media reports after the final data reading. Fifth row: Media is kept constant until the declared pandemic end, week 47, then cut off.

**Fig 6:**
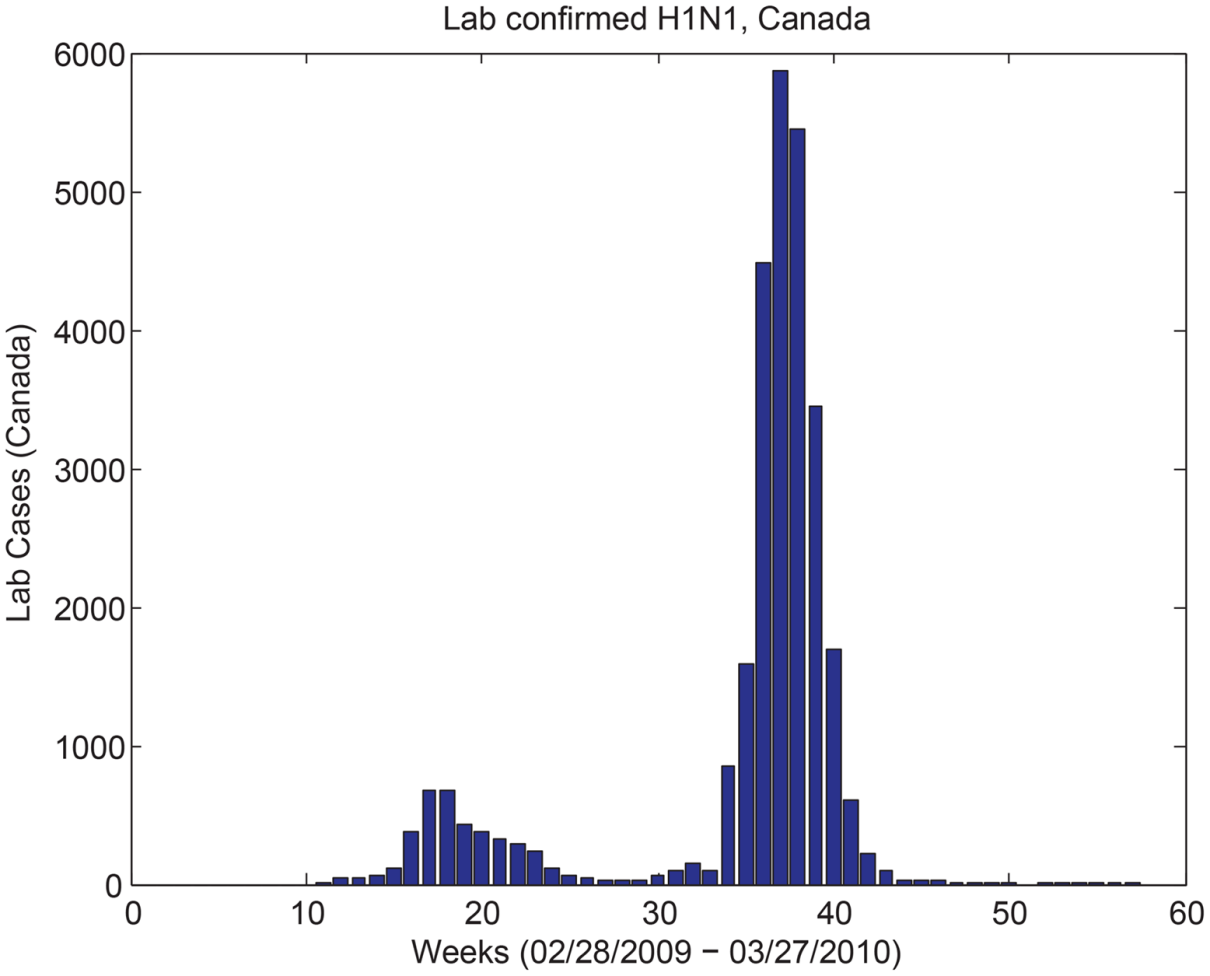
H1N1 cases. Lab confirmed cases of pandemic H1N1 in 2009 in Canada [44]. There are two waves of the pandemic.

In the previous sections is was assumed that the mass media level of reporting was proportional to the number of report symptomatic cases, related to a fraction of incoming infectious cases *ρσE*. The structure of Eq (3), however, allows for the incorporation of real mass media data into the model like that compiled by GPHIN (Fig 3) [34]. Fig 5 shows the results of Eq (3) when social distancing and waning behaviour are included in the model with (right column) and without (left column) vaccination and the media data [34]. Here, the English language media report levels are used (see Fig 3, dashed line) (top row), and the media data is then extended to consider various scenarios of media reporting until the end of the pandemic: a constant level equal to the last collected data point (second row), a decay after the final data point (third row), setting the report level to zero at the end of the data set (fourth row), and setting the number of reports to zero at the declared end of the pandemic (week 47 [44]). Note that when vaccination is included in the model (right column), the vaccination rate for each of the scenarios is between 25% to 40% of the population [2], and vaccination is included only in the second wave of infection. Fig 5 demonstrates that for all cases, two waves of the pandemic occur, with the magnitude of the peaks varying by the model assumptions. Interestingly, these dynamics agree with what was observed in many countries globally during the 2009 H1N1 pandemic—two waves of infection with two distinct peaks were observed in this timeframe. For a qualitative comparison we plot the total number of confirmed cases in Canada during the 2009 H1N1 pandemic [44] that shows this two wave behaviour (see Fig 6).

Fig 5 demonstrates that for all cases, the conclusion of the pandemic occurs in a similar time frame, but the shortest pandemic time results from keeping the number of reports constant until the infection is cleared from the population (second row). The similar pandemic curves for each scenario, even with a change in media behaviour at the end of the GPHIN data could be due to the fact that the pandemic is almost finished by the time the last media point is collected. i.e., there are not very many individuals left in the population that have not been affected by the pandemic.

In addition to English, French is another official language in Canada. When the French language reports are included in the model similar results are found—two peaks of infection occur, however, there is a smaller number of total infections due to the fact that there are more media reports used in the media equation and the total population N being considered was unchanged.

A further simulation considering the same population size and all media reports in all languages (Fig 3 solid line) results in a a single small wave of infection (not shown). Again, this is an intuitive result since the model assumes that if there are more media reports, more individuals will uptake social distancing practices and vaccination. However, if we increase the total population size, which better represents a population that is affected by media reports in all of the languages tracked by GPHIN, two peaks of infection will result.

## Discussion

Mathematical modelling studies of the effects of mass media on an epidemic either include a specific function that decreases the transmission rate or a mass media compartment [19–32]. In a recent study, we demonstrated that epidemic outcomes can vary greatly depending on the mass media function chosen, making it difficult to determine any quantification of the variability in key epidemic measurements for public health [33]. It was concluded that models that include a mass media compartment should thus be employed [33]. Mathematical models that include a mass media compartment also enable the study of media fatigue, and can be further modified to include actual mass media report data. In this study we developed a model that includes a media compartment.

Eq (3) includes a mass media compartment, vaccination and two susceptible classes that practice social distancing at different levels. The model also includes the effects of media fatigue and waning social distancing practices. Table 2 demonstrates that social distancing and vaccination benefit a population (a decreased level of infection results). This table also shows, however, that media fatigue and waning behaviour can lessen the positive benefits of these intervention measures (last column).

An agent-based Monte Carlo simulation based on Eq (3) was employed to study the variability in key epidemic measurements that must be considered in public health policies and planning. Results showed that the expected outcome of the simulations is quantitatively similar to the solution of deterministic system of ordinary differential equations of Eq (3), but that, variability in the key epidemic measurements can be quite large (Table 2). This in turn will affect public health resources and planning.

A sensitivity analysis of Eq (3) demonstrated that the parameters associated with mass media reports and media fatigue can significantly affect epidemic outcomes. Thus, more time and effort should be given to the study of the effects of these reports during epidemics/pandemics so that these effects can be accounted for in public health policies and preparedness plans.

In the “Mass Media Data and Media Waning” section we incorporated mass media data, compiled by the Global Public Health Intelligence Network [16, 34], into Eq (3). This study demonstrated that two waves of infection can occur when the GPHIN mass media data is included in the model. This is an interesting result for two reasons. First, two waves of infection were experienced by many countries globally during the 2009 H1N1 pandemic, including Canada. Second, previous studies of the two wave behaviour have mainly focused on the effects of school holidays during the pandemic, which effectively reduced transmission and then increased it again a couple months later, producing two waves of infection [44–47]. Here, we have shown that media fatigue can also produce two waves of infection with similar qualitative dynamics to that observed over the 2009 H1N1 pandemic.

As stated above, models incorporating school closure over the summer months can generate two waves of infection [47]. Differences in mixing between age groups, demography and mobility [48], and now, mass media reports and waning behaviour can also contribute to the two wave phenomenon. It is of interest to determine what the magnitude of these contributions to the double peak behaviour can be. This is a course for future work. This would also lead to a study of the optimal use of media reporting in public health [32, 49].

Eq (3) includes two levels of social distancing in the susceptible classes. However, social distancing behaviours can be interpreted to occur over a continuous scale from moderate to intensive levels of social distancing. Also, exposed and infectious individuals can also practice social distancing measures. For example, an infected individual may elect to stay home from work, and distance themselves from family members in the same household as well. To model continuous social distancing in all classes we propose a new model employing partial differential equations that keeps track of all classes of infection over time and social distancing space. Analysis of this model is current work. In future work, the model will be augmented to include asymptomatic infections, where individuals are infectious but do not demonstrate symptoms. This will affect transmission, as social distancing will not be enhanced if infection is asymptomatic, and thus, mass media messages that encourage social distancing will be ever more important.

Mass media messages can relay information that will induce positive or negative social distancing practices. Mass media reports can also affect vaccination uptake. For example, the reporting of the benefits and risks may induce vaccination uptake in some individuals, but induce vaccine hesitancy in others. A study of the effects of the type of mass media message on social distancing behaviour is greatly needed, and is a course for future work.

Finally, in this study we focused our work on understanding the effects of mass media data and media fatigue in the context of the 2009 H1N1 pandemic. Similar studies of other infectious diseases would also be of interest. MERS-CoV and Ebola in the current day are a specific interest.

## Appendix

### Local stability of the disease free equilibrium *E*_0_

**Theorem 0.1** *The disease free equilibrium E_0_ is locally asymptotically stable if R_0_* < 1 *and unstable if R_0_* > 1.

The Jacobian matrix for model Eq (3) evaluated at the DFE *E*_0_ is

$$\begin{array}{c} \left[\begin{array}{cccccccc} -d & q_{1} & 0 & 0 & 0 & -\beta \;S_{0} & 0 & -\alpha \;S_{0}-\nu S_{0}\\ 0 & -q_{1}-d & q_{2} & 0 & 0 & -\beta_{1}S_{0} & 0 & \alpha S_{0}\\ 0 & 0 & -d-q_{2} & 0 & 0 & 0 & 0 & 0\\ 0 & 0 & 0 & -d & 0 & 0 & 0 & \nu S_{0}\\ 0 & 0 & 0 & 0 & -\sigma -d & \beta S_{0} & 0 & 0\\ 0 & 0 & 0 & 0 & \sigma & -\gamma -d & 0 & 0\\ 0 & 0 & 0 & 0 & 0 & \gamma & -d & 0\\ 0 & 0 & 0 & 0 & \rho \sigma & 0 & 0 & -\rho_{1} \end{array}\right] \end{array}$$ (9)

where *S*_0_ = *λ*/*d*. The eigenvalues *ϕ* corresponding to the Jacobian matrix evaluated at *E*_0_ are:

$$\begin{array}{ccc} \phi_{1,2,3} & = & -d,\;\phi_{4}=-\left(q_{1}-+d\right),\;\phi_{5}=-\left(q_{2}+d\right),\;\phi_{6}=-\rho_{1},\\ \phi_{7,8} & = & \frac{-\sigma -2d-\gamma \pm \sqrt{\sigma^{2}-2\sigma \gamma +\gamma^{2}+4\sigma \beta S_{0}}}{2}. \end{array}$$ (10)

The local stability of *E*_0_ thus depends solely on the values of *ϕ*_7,8_. Rearranging *ϕ*_7,8_ < 0 we obtain

$$\begin{array}{ccc} \frac{\sigma \beta \lambda }{\sigma \gamma d+\sigma d^{2}+\gamma d^{2}+d^{3}} & < & 1\\ \frac{\sigma \beta \lambda }{d(\sigma +d)(\gamma +d)} & < & 1\\ R_{0} & < & 1. \end{array}$$ (11)

Thus, *E*_0_ is locally asymptotically stable if *R*_0_ < 1 and unstable if *R*_0_ > 1.

### Stochastic differential equations

As an example, we now derive a system of stochastic differential equations for the standard SEIR model

$$\begin{array}{c} \begin{array}{ccc} \frac{dS}{dt} & = & \lambda -\beta SI-dS\\ \frac{dE}{dt} & = & \beta SI-\sigma E-dE\\ \frac{dI}{dt} & = & \sigma E-\gamma I-dI\\ \frac{dR}{dt} & = & \gamma I-dR \end{array} \end{array}$$ (12)

as derived in [50]. We construct a stochastic differential equation model from the continuous time Markov chain (CTMC) model for an SEIR model by considering time as continuous on *tε*[0, ∞), the collection of discrete random variables *S*(*t*), *E*(*t*), *I*(*t*), and *R*(*t*) for susceptible, exposed, infectious and recovered classes or states, respectively. Transition probability from state to state is called the infinitesimal transition probability, the change in time is sufficiently small that only one event occurs in the time period, and depends only on the state of the system at the previous time: Markov property. The probability of a transition can be described by Eq (13),

$$\begin{array}{c} Prob\{\Delta S(t)=i,\Delta E(t)=j,\Delta I(t)=k,\Delta R(t)=l|S(t),E(t),I(t),R(t)\}, \end{array}$$ (13)

where Δ*S*(*t*) = *S*(*t* + Δ*t*) − *S*(*t*). Each of *i*, *j*, *k*, *l* take on values of only + 1, −1 or 0, because only one change occurs in sufficiently small Δ*t*, thus *o*(Δ*t*) is included in the definition of the infinitesimal transition probabilities. The probabilities for each change in state are straightforward to describe from an SEIR model and can be seen in Eq (14).

$$\begin{array}{c} Prob\left\{\Delta S\left(t\right)=i,\Delta E\left(t\right)=j,\Delta I\left(t\right)=k,\Delta R\left(t\right)=l|S\left(t\right),E\left(t\right),I\left(t\right),R\left(t\right)\right\}=\{\begin{array}{c} \lambda \Delta t+o(\Delta t),\;(i,j,k,l)=(1,0,0,0)\\ \beta S(t)I(t)+o(\Delta t),\;(i,j,k,l)=(-1,1,0,0)\\ dS(t)+o(\Delta t),\;(i,j,k,l)=(-1,0,0,0)\\ \sigma E(t)+o(\Delta t),\;(i,j,k,l)=(0,-1,1,0)\\ dE(t)+o(\Delta t),\;(i,j,k,l)=(0,-1,0,0)\\ \gamma I(t)+o(\Delta t),\;(i,j,k,l)=(0,0,-1,1)\\ dI(t)+o(\Delta t),\;(i,j,k,l)=(0,0,-1,0)\\ dR(t)+o(\Delta t),\;(i,j,k,l)=(0,0,0,-1)\\ (1-(\lambda +\beta S(t)I(t)+dS(t)+\sigma E(t)+\\ dE(t)+\gamma I(t)+dI(t)+dR(t)))\Delta t+o(\Delta t),\;(i,j,k,l)=(0,0,0,0)\\ o(\Delta t),\;\text{otherwise.} \end{array} \end{array}$$ (14)

From the equations for the infinitesimal transition probabilities, Eq (14), we can derive the forward Kolmogorov differential equations. From the forward Kolmogorov differential equation we derive a probability generating function (pgf), a moment generating function (mgf) and finally the differential equations for the mean and higher order moments.

The derivation for the forward Kolmogorov differential equation from Eq (14), representing the rate of change of the transitions from state to state follows. The first step is to derive a discrete equation from Eq (14) for sufficiently small Δ*t* for *p*_*i*,*j*,*k*,*l*_(*t* + Δ*t*), the transition probability,

$$\begin{array}{ccc} p_{i,j,k,l}\left(t+\Delta t\right) & = & \lambda p_{i-1,j,k,l}\left(t\right)\Delta t+\beta \left(i+1\right)kp_{i+1,j-1,k,l}\left(t\right)\Delta t\\ & & +d\left(i+1\right)p_{i+1,j,k,l}\Delta t+\sigma \left(j+1\right)p_{i,j+1,k-1,l}\left(t\right)\Delta t\\ & & +d\left(j+1\right)p_{i,j+1,k,l}\left(t\right)\Delta t+\gamma \left(k+1\right)p_{i,j,k+1,l-1}\left(t\right)\Delta t\\ & & +d\left(k+1\right)p_{i,j,k+1,l}\left(t\right)\Delta t+d\left(l+1\right)p_{i,j,k,l+1}\left(t\right)\Delta t\\ & & +\left(1-\left(\lambda +\beta ik+di+\sigma j+dj+\gamma k+dk+dl\right)\right)p_{i,j,k,l}\left(t\right)\Delta t, \end{array}$$ (15)

where *p*_*i*,*j*,*k*,*l*_(*t*) represents the probability of a susceptible, exposed, infectious and recovered population of size *i*, *j*, *k*, and *l*, respectively. Next, *p*_*i*,*j*,*k*,*l*_(*t*) is subtracted from both sides, the expressions are divided by Δ*t*, resulting in Eq (16),

$$\begin{array}{ccc} \frac{p_{i,j,k,l}\left(t+\Delta t\right)-p_{i,j,k,l}\left(t\right)}{\Delta t} & = & \sum [\lambda p_{i-1,j,k,l}\left(t\right)S^{i}E^{j}I^{k}R^{l}+\beta \left(i+1\right)kp_{i+1,j-1,k,l}\left(t\right)S^{i}E^{j}I^{k}R^{l}\\ & & +d\left(i+1\right)p_{i+1,j,k,l}S^{i}E^{j}I^{k}R^{l}\\ & & +\sigma \left(j+1\right)p_{i,j+1,k-1,l}\left(t\right)S^{i}E^{j}I^{k}R^{l}+d\left(j+1\right)p_{i,j+1,k,l}\left(t\right)S^{i}E^{j}I^{k}R^{l}\\ & & +\gamma \left(k+1\right)p_{i,j,k+1,l-1}\left(t\right)S^{i}E^{j}I^{k}R^{l}+d\left(k+1\right)p_{i,j,k+1,l}\left(t\right)S^{i}E^{j}I^{k}R^{l}\\ & & +d\left(l+1\right)p_{i,j,k,l+1}\left(t\right)S^{i}E^{j}I^{k}R^{l}\\ & & -\lambda p_{i,j,k,l}\left(t\right)S^{i}E^{j}I^{k}R^{l}-\beta ikp_{i,j,k,l}\left(t\right)S^{i}E^{j}I^{k}R^{l}\\ & & -dip_{i,j,k,l}\left(t\right)S^{i}E^{j}I^{k}R^{l}-\sigma jp_{i,j,k,l}\left(t\right)S^{i}E^{j}I^{k}R^{l}\\ & & -djp_{i,j,k,l}\left(t\right)S^{i}E^{j}I^{k}R^{l}-\gamma kp_{i,j,k,l}\left(t\right)S^{i}E^{j}I^{k}R^{l}\\ & & -dkp_{i,j,k,l}\left(t\right)S^{i}E^{j}I^{k}R^{l}-dlp_{i,j,k,l}\left(t\right)S^{i}E^{j}I^{k}R^{l}]\\ & & +\frac{o(\Delta t}{\Delta t}. \end{array}$$ (16)

Next take sum over (*i*, *j*, *k*, *l*) and take lim Δ*t* → 0 and arrive at the forward Kolmogorov differential equation, Eq (17),

$$\begin{array}{ccc} \frac{dp_{i,j,k,l}\left(t\right)}{dt} & = & \lambda p_{i-1,j,k,l}\left(t\right)S^{i}E^{j}I^{k}R^{l}+\beta \left(i+1\right)kp_{i+1,j-1,k,l}\left(t\right)S^{i}E^{j}I^{k}R^{l}\\ & & +d\left(i+1\right)p_{i+1,j,k,l}S^{i}E^{j}I^{k}R^{l}\\ & & +\sigma \left(j+1\right)p_{i,j+1,k-1,l}\left(t\right)S^{i}E^{j}I^{k}R^{l}+d\left(j+1\right)p_{i,j+1,k,l}\left(t\right)S^{i}E^{j}I^{k}R^{l}\\ & & +\gamma \left(k+1\right)p_{i,j,k+1,l-1}\left(t\right)S^{i}E^{j}I^{k}R^{l}+d\left(k+1\right)p_{i,j,k+1,l}\left(t\right)S^{i}E^{j}I^{k}R^{l}\\ & & +d\left(l+1\right)p_{i,j,k,l+1}\left(t\right)S^{i}E^{j}I^{k}R^{l}\\ & & -\lambda p_{i,j,k,l}\left(t\right)S^{i}E^{j}I^{k}R^{l}-\beta ikp_{i,j,k,l}\left(t\right)S^{i}E^{j}I^{k}R^{l}\\ & & -dip_{i,j,k,l}\left(t\right)S^{i}E^{j}I^{k}R^{l}-\sigma jp_{i,j,k,l}\left(t\right)S^{i}E^{j}I^{k}R^{l}\\ & & -djp_{i,j,k,l}\left(t\right)S^{i}E^{j}I^{k}R^{l}-\gamma kp_{i,j,k,l}\left(t\right)S^{i}E^{j}I^{k}R^{l}\\ & & -dkp_{i,j,k,l}\left(t\right)S^{i}E^{j}I^{k}R^{l}-dlp_{i,j,k,l}\left(t\right)S^{i}E^{j}I^{k}R^{l}. \end{array}$$ (17)

Next, replace the deterministic variables *i*, *j*, *k*, *l* with the state variables of *S*, *E*, *I*, *R* to arrive at the probability generating function (pgf) Eq (18)

$$\begin{array}{ccc} \frac{dP}{dt} & = & \lambda \left(S-1\right)P\left(S,E,I,R,t\right)+\beta I\left(E-S\right)\frac{\partial^{2}P}{\partial S\partial I}+\sigma \left(I-E\right)\frac{\partial P}{\partial E}\\ & & +d\left(1-S\right)\frac{\partial P}{\partial S}+d\left(1-E\right)\frac{\partial P}{\partial E}+\gamma \left(R-I\right)\frac{\partial P}{\partial I}\\ & & +d\left(1-I\right)\frac{\partial P}{\partial I}+d\left(1-R\right)\frac{\partial P}{\partial R}. \end{array}$$ (18)

Here, convert the pgf to the mgf, since the method of derivation of the moment differential equations is simpler from the moment generating function, defined as *M*(*θ*, *ϕ*, *ψ*, *ζ*, *t*) = *P*(*S*, *E*, *I*, *R*, *t*)*e* ^*Sθ* + *Eϕ* + *Iψ* + *Rζ*^. From the probability generating function (pgf), take the total derivative of *M*(*θ*, *ϕ*, *ψ*, *ζ*, *t*) to get function Eq (19).

$$\begin{array}{ccc} \frac{\partial M}{\partial t} & = & \lambda \left(e^{\theta }-1\right)M+\beta \left(e^{\phi -\theta }-1\right)\frac{\partial^{2}M}{\partial \theta \partial \psi }+d\left(e^{-\theta }-1\right)\frac{\partial M}{\partial \theta }\\ & & +\sigma \left(e^{\psi -\phi }-1\right)\frac{\partial M}{\partial \phi }+d\left(e^{-\phi }-1\right)\frac{\partial M}{\partial \phi }+\gamma \left(e^{\zeta -\psi }-1\right)\frac{\partial M}{\partial \psi }\\ & & +d\left(e^{-\psi }-1\right)\frac{\partial M}{\partial \psi }+d\left(e^{-\zeta }-1\right)\frac{\partial M}{\partial \zeta }. \end{array}$$ (19)

To derive the system of stochastic differential equations for *S*, *E*, *I*, and *R*, take the derivative of Eq (19) with respect to the variable that corresponds to the moment required for the differential equation; for example, if *E*[*S*] is required, take the partial derivative of Eq (19) w.r.t *θ* and evaluate the results at (*θ*, *ϕ*, *ψ*, *ζ*) = (0,0,0,0),

$$\begin{array}{c} \frac{dE[S]}{dt}=\lambda -\beta E\left[SI\right]-dE\left[S\right], \end{array}$$ (20)

depending on the higher order moment *E*[*SI*] = *E*[*S*]*E*[*I*] + *cov*(*S*, *I*). If *cov*(*S*, *I*) = 0, the ordinary differential equation is returned and the state variables of the system are independent. Obviously this cannot be solved explicitly, as it is not closed, differential equations for the higher order moments must be considered, in an attempt to solve the equation. Take the derivative of Eq (19) w.r.t *S* and *I*, and end up with

$$\begin{array}{c} \frac{d(E[SI])}{dt}=\lambda E\left[I\right]-\beta E\left[IIS\right]-2dE\left[SI\right]+\sigma E\left[SE\right]-\gamma E\left[SI\right]. \end{array}$$ (21)

It is obvious that this equation contains the third order moment. The system of stochastic differential equations is

$$\begin{array}{ccc} \frac{dE[S]}{dt} & = & \lambda -\beta E[SI]-dE[S]\\ \frac{dE[E]}{dt} & = & \beta E[SI]-\sigma E[E]-dE[E]\\ \frac{dE[I]}{dt} & = & \sigma E[E]-\gamma E[I]-dE[I]\\ \frac{dE[R]}{dt} & = & \gamma E[I]-dE[R]\\ \frac{dE[SS]}{dt} & = & \lambda +2\lambda E[S]+\beta E[SI]-2\beta E[SSI]+dE[S]-2dE[SS]\\ \frac{dE[EE]}{dt} & = & \beta E[SI]+2\beta E[SEI]+\sigma E[E]-2\sigma E[EE]+dE[E]-2dE[EE]\\ \frac{dE[II]}{dt} & = & \sigma E[E]+2\sigma E[EI]+\gamma E[I]-2\gamma E[II]+dE[I]-2dE[II]\\ \frac{dE[SI]}{dt} & = & \lambda E[I]-\beta E[IIS]-2dE[SI]+\sigma E[SE]-\gamma E[SI]\\ \frac{dE[EI]}{dt} & = & \beta E[IIS]-\sigma E[E]-\sigma E[EI]+\sigma E[EE]-2dE[EI]-\gamma E[EI]\\ \frac{dE[SE]}{dt} & = & \lambda E[E]-\beta E[SI]-\beta E[SEI]+\beta E[SSI]-2dE[SE]-\sigma E[SE]. \end{array}$$ (22)

To choose which higher order moment equations to explicitly define, look at which 2^nd^ order moments appear in the equations of the first order moments. Next look at which equations of 2nd order moments appear in the already existing 2nd order moment equations. Since there are no 2nd order moments involving *E*[*R*], there is no 2nd order moment equation for *E*[*R*].

The solution of Eq (22) is almost identical to the solution of Eq (3) (not shown). Thus, for the purposes of this study we move forward considering Eq (3) only.
